# (*E*)-4-Octyloxybenzaldehyde thio­semicarbazone

**DOI:** 10.1107/S1600536809054841

**Published:** 2009-12-24

**Authors:** M. A. A. A. A. Islam, M. T. H. Tarafder, C. M. Zakaria, N. Guidolin, E. Zangrando

**Affiliations:** a Department of Chemistry, Rajshahi University of Engineering & Technology, Rajshahi 6204, Bangladesh; b Department of Chemistry, Rajshahi University, Rajshahi 6205, Bangladesh; c Dipartimento di Scienze Chimiche, Via Licio Giorgieri 1, 34127 Trieste, Italy

## Abstract

In the title compound, C_16_H_25_N_3_OS, the thio­semicarbazone group adopts an *E* configuration with respect to the C=N bond and is almost coplanar with the benzene ring, forming a dihedral angle of 9.3 (1)°. In the crystal packing, the mol­ecules lie along the *a* axis in an anti­parallel arrangement and are held in place by van der Waals inter­actions. As a consequence, there is relatively low anisotropic thermal motion in the terminal atoms of the *n*-octyl chain.

## Related literature

For the related structures, see: Basuli *et al.* (2000 ▶); Narayana *et al.* (2007 ▶); Pal *et al.* (2002 ▶); Tian *et al.* (2002 ▶); Tarafder *et al.* (2008 ▶).
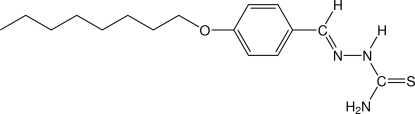


## Experimental

### 

#### Crystal data

C_16_H_25_N_3_OS
*M* *_r_* = 307.45
Triclinic, 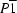

*a* = 5.785 (2) Å
*b* = 7.586 (2) Å
*c* = 20.789 (4) Å
α = 94.74 (2)°
β = 91.85 (2)°
γ = 104.42 (3)°
*V* = 879.2 (4) Å^3^
*Z* = 2
Mo *K*α radiation
μ = 0.19 mm^−1^
*T* = 293 K
0.42 × 0.40 × 0.14 mm

#### Data collection

Enraf–Nonius dip1030 image-plate diffractometer
9930 measured reflections
3226 independent reflections
2749 reflections with *I* > 2σ(*I*)
*R*_int_ = 0.030

#### Refinement

*R*[*F* ^2^ > 2σ(*F* ^2^)] = 0.045
*wR*(*F* ^2^) = 0.140
*S* = 1.04
3226 reflections
191 parameters
H-atom parameters constrained
Δρ_max_ = 0.19 e Å^−3^
Δρ_min_ = −0.18 e Å^−3^

### 

Data collection: *XPRESS* (MacScience, 2002 ▶); cell refinement: *DENZO* (Otwinowski & Minor, 1997 ▶); data reduction: *DENZO* and *SCALEPACK* (Otwinowski & Minor, 1997 ▶); program(s) used to solve structure: *SHELXS97* (Sheldrick, 2008 ▶); program(s) used to refine structure: *SHELXL97* (Sheldrick, 2008 ▶); molecular graphics: *ORTEP-3 for Windows* (Farrugia, 1997 ▶); software used to prepare material for publication: *WinGX* (Farrugia, 1999 ▶).

## Supplementary Material

Crystal structure: contains datablocks global, I. DOI: 10.1107/S1600536809054841/fj2264sup1.cif

Structure factors: contains datablocks I. DOI: 10.1107/S1600536809054841/fj2264Isup2.hkl

Additional supplementary materials:  crystallographic information; 3D view; checkCIF report

## Figures and Tables

**Fig. 1.**
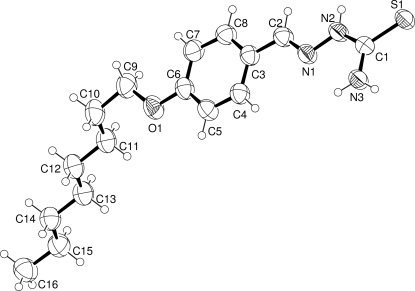
ORTEP drawing (ellipsoids at the 40% probability level) of the compoud with atom-labelling scheme.

## supplementary crystallographic information

### Comment

When used in coordination chemistry this molecule is potentially a bidentate ligand acting through the α- or β-nitrogen and thiolate sulfur anion forming a four- and five-membered chelate ring, respectively (Pal *et al.*, 2002), although a behavior as monocoordinated ligand through sulfur has also been reported (Tian *et al.*, 2002). In the crystal structure the molecules are interconnected by N—H···N and N–H···S hydrogen bonds as found in other thiosemicarbazone species (Narayana *et al.*, 2007; Tarafder *et al.*, 2008). The crystal structure is also stabilized by C–H···π interactions. The octyl chain presents an anti conformation with the exception of the O1—C9—C10—C11 part that has a torsion angle of -72.7 (3)°.

### Experimental

4-n-octyloxybenzaldehyde (6.09 g, 26 mmol) was added to a hot solution of thiosemicarbazide (2.38 g, 26 mmol) in methanol (200 ml). The mixture was refluxed for 30 min and cooled down to room temperature. The product was recrystallized from dichloromethane to give colorless microcrystals. M.P. 381 K. Brilliant colorless flat rectangular shaped crystals suitable for X-ray difraction were obtained from a mixture of dichloromethane and toluene (10:5; *v*/*v*) after 5 days.

### Refinement

Data collection was performed on a image plate with a phi scan over 180° that allows to get a completion (for the triclinic space group) of 97%. All H atoms were located geometrically and treated as riding atoms, with C—H = 0.93–0.96 Å, N—H = 0.86 and with *U*ĩso~(H) = 1.2*U*~eq~(C or N) or 1.5*U*~eq~(C) for methyl H atoms.

### Crystal data

**Table:**

C_16_H_25_N_3_OS	*Z* = 2
*M**_r_* = 307.45	*F*(000) = 332
Triclinic, *P*1	*D*_x_ = 1.161 Mg m^−^^3^
Hall symbol: -P 1	Mo *K*α radiation, λ = 0.71073 Å
*a* = 5.785 (2) Å	Cell parameters from 146 reflections
*b* = 7.586 (2) Å	θ = 3.0–18.1°
*c* = 20.789 (4) Å	µ = 0.19 mm^−^^1^
α = 94.74 (2)°	*T* = 293 K
β = 91.85 (2)°	Plate, colorless
γ = 104.42 (3)°	0.42 × 0.40 × 0.14 mm
*V* = 879.2 (4) Å^3^	

### Data collection

**Table:**

Enraf–Nonius dip1030 image-plate diffractometer	2749 reflections with *I* > 2σ(*I*)
Radiation source: fine-focus sealed tube	*R*_int_ = 0.030
graphite	θ_max_ = 25.7°, θ_min_ = 3.6°
φ–scans with narrow frames	*h* = −7→7
9930 measured reflections	*k* = −9→8
3226 independent reflections	*l* = −25→25

### Refinement

**Table:**

Refinement on *F*^2^	Primary atom site location: structure-invariant direct methods
Least-squares matrix: full	Secondary atom site location: difference Fourier map
*R*[*F*^2^ > 2σ(*F*^2^)] = 0.045	Hydrogen site location: inferred from neighbouring sites
*wR*(*F*^2^) = 0.140	H-atom parameters constrained
*S* = 1.04	*w* = 1/[σ^2^(*F*_o_^2^) + (0.1026*P*)^2^ + 0.0242*P*] where *P* = (*F*_o_^2^ + 2*F*_c_^2^)/3
3226 reflections	(Δ/σ)_max_ < 0.001
191 parameters	Δρ_max_ = 0.19 e Å^−^^3^
0 restraints	Δρ_min_ = −0.18 e Å^−^^3^

### Special details

**Table:**

Geometry. All s.u.'s (except the s.u. in the dihedral angle between two l.s. planes) are estimated using the full covariance matrix. The cell s.u.'s are taken into account individually in the estimation of s.u.'s in distances, angles and torsion angles; correlations between s.u.'s in cell parameters are only used when they are defined by crystal symmetry. An approximate (isotropic) treatment of cell s.u.'s is used for estimating s.u.'s involving l.s. planes.
Refinement. Refinement of *F*^2^ against ALL reflections. The weighted *R*-factor *wR* and goodness of fit *S* are based on *F*^2^, conventional *R*-factors *R* are based on *F*, with *F* set to zero for negative *F*^2^. The threshold expression of *F*^2^ > 2σ(*F*^2^) is used only for calculating *R*-factors(gt) *etc*. and is not relevant to the choice of reflections for refinement. *R*-factors based on *F*^2^ are statistically about twice as large as those based on *F*, and *R*- factors based on ALL data will be even larger.

### Fractional atomic coordinates and isotropic or equivalent isotropic displacement parameters (Å^2^)

**Table:**

	*x*	*y*	*z*	*U*_iso_*/*U*_eq_	
S1	1.33919 (6)	−0.29398 (5)	0.99374 (2)	0.06930 (19)	
N1	1.0386 (2)	0.02579 (17)	0.89975 (6)	0.0648 (3)	
N2	1.1983 (2)	−0.04336 (17)	0.93483 (6)	0.0690 (3)	
H2	1.3435	0.0203	0.9421	0.083*	
N3	0.9025 (2)	−0.29670 (17)	0.94909 (6)	0.0668 (3)	
H3A	0.8033	−0.2489	0.9295	0.080*	
H3B	0.8534	−0.4024	0.9631	0.080*	
O1	0.6110 (2)	0.55328 (16)	0.73145 (6)	0.0812 (3)	
C1	1.1305 (2)	−0.20768 (19)	0.95761 (7)	0.0591 (3)	
C2	1.1216 (3)	0.1882 (2)	0.88465 (7)	0.0663 (4)	
H2A	1.2764	0.2498	0.8995	0.080*	
C3	0.9864 (3)	0.2818 (2)	0.84546 (7)	0.0630 (4)	
C4	0.7501 (3)	0.2031 (2)	0.82202 (7)	0.0681 (4)	
H4	0.6725	0.0867	0.8321	0.082*	
C5	0.6322 (3)	0.2971 (2)	0.78409 (8)	0.0716 (4)	
H5	0.4761	0.2431	0.7683	0.086*	
C6	0.7444 (3)	0.4721 (2)	0.76919 (7)	0.0677 (4)	
C7	0.9777 (3)	0.5528 (2)	0.79263 (7)	0.0726 (4)	
H7	1.0536	0.6704	0.7833	0.087*	
C8	1.0957 (3)	0.4563 (2)	0.82997 (7)	0.0698 (4)	
H8	1.2526	0.5100	0.8451	0.084*	
C9	0.7089 (4)	0.7404 (2)	0.72041 (9)	0.0880 (5)	
H9A	0.8564	0.7529	0.6983	0.106*	
H9B	0.7432	0.8161	0.7612	0.106*	
C10	0.5257 (4)	0.7986 (3)	0.67937 (9)	0.0890 (5)	
H10A	0.3717	0.7624	0.6982	0.107*	
H10B	0.5693	0.9309	0.6805	0.107*	
C11	0.5012 (4)	0.7192 (3)	0.60985 (8)	0.0811 (5)	
H11A	0.4523	0.5869	0.6085	0.097*	
H11B	0.6561	0.7522	0.5913	0.097*	
C12	0.3221 (3)	0.7838 (3)	0.56889 (9)	0.0817 (5)	
H12A	0.1660	0.7448	0.5863	0.098*	
H12B	0.3662	0.9164	0.5727	0.098*	
C13	0.3025 (3)	0.7155 (2)	0.49799 (9)	0.0826 (5)	
H13A	0.2573	0.5828	0.4940	0.099*	
H13B	0.4583	0.7543	0.4803	0.099*	
C14	0.1228 (3)	0.7824 (3)	0.45814 (8)	0.0810 (5)	
H14A	−0.0331	0.7420	0.4756	0.097*	
H14B	0.1667	0.9150	0.4629	0.097*	
C15	0.1029 (4)	0.7182 (3)	0.38689 (9)	0.0891 (5)	
H15A	0.0562	0.5857	0.3819	0.107*	
H15B	0.2589	0.7573	0.3693	0.107*	
C16	−0.0752 (4)	0.7893 (3)	0.34816 (10)	0.0987 (6)	
H16A	−0.2329	0.7416	0.3624	0.148*	
H16B	−0.0710	0.7509	0.3031	0.148*	
H16C	−0.0347	0.9204	0.3544	0.148*	

### Atomic displacement parameters (Å^2^)

**Table:**

	*U*^11^	*U*^22^	*U*^33^	*U*^12^	*U*^13^	*U*^23^
S1	0.0526 (3)	0.0696 (3)	0.0871 (3)	0.01517 (17)	−0.00582 (18)	0.02038 (19)
N1	0.0600 (7)	0.0703 (8)	0.0671 (7)	0.0211 (5)	−0.0045 (5)	0.0120 (6)
N2	0.0551 (7)	0.0706 (8)	0.0817 (8)	0.0141 (6)	−0.0097 (6)	0.0204 (6)
N3	0.0527 (6)	0.0681 (7)	0.0810 (8)	0.0158 (5)	−0.0032 (5)	0.0155 (6)
O1	0.0863 (8)	0.0834 (7)	0.0823 (7)	0.0315 (6)	−0.0029 (6)	0.0277 (6)
C1	0.0534 (7)	0.0639 (8)	0.0604 (8)	0.0158 (6)	0.0003 (6)	0.0060 (6)
C2	0.0666 (9)	0.0705 (9)	0.0627 (8)	0.0181 (7)	−0.0032 (6)	0.0120 (7)
C3	0.0680 (8)	0.0687 (8)	0.0555 (7)	0.0224 (6)	0.0007 (6)	0.0090 (6)
C4	0.0709 (9)	0.0664 (8)	0.0691 (9)	0.0191 (7)	0.0004 (7)	0.0152 (7)
C5	0.0677 (9)	0.0792 (10)	0.0713 (9)	0.0228 (7)	−0.0027 (7)	0.0159 (7)
C6	0.0774 (9)	0.0744 (9)	0.0587 (8)	0.0309 (7)	0.0027 (7)	0.0132 (7)
C7	0.0831 (10)	0.0666 (9)	0.0687 (9)	0.0177 (7)	0.0007 (7)	0.0153 (7)
C8	0.0712 (9)	0.0711 (9)	0.0662 (9)	0.0159 (7)	−0.0042 (7)	0.0102 (7)
C9	0.1125 (15)	0.0781 (11)	0.0813 (11)	0.0368 (10)	−0.0047 (10)	0.0185 (9)
C10	0.1121 (15)	0.0853 (11)	0.0837 (12)	0.0468 (11)	0.0025 (10)	0.0239 (9)
C11	0.0900 (12)	0.0816 (11)	0.0815 (11)	0.0349 (9)	0.0066 (9)	0.0225 (8)
C12	0.0882 (12)	0.0861 (11)	0.0802 (11)	0.0350 (9)	0.0053 (9)	0.0224 (9)
C13	0.0886 (12)	0.0810 (11)	0.0843 (11)	0.0299 (9)	0.0039 (9)	0.0174 (9)
C14	0.0875 (12)	0.0802 (10)	0.0797 (11)	0.0276 (9)	0.0040 (8)	0.0146 (8)
C15	0.0945 (13)	0.0900 (12)	0.0859 (12)	0.0307 (10)	−0.0021 (9)	0.0068 (9)
C16	0.1001 (14)	0.1156 (15)	0.0842 (12)	0.0372 (12)	−0.0079 (10)	0.0060 (11)

### Geometric parameters (Å, °)

**Table:**

S1—C1	1.6930 (15)	C9—H9A	0.9700
N1—C2	1.275 (2)	C9—H9B	0.9700
N1—N2	1.3863 (16)	C10—C11	1.507 (3)
N2—C1	1.341 (2)	C10—H10A	0.9700
N2—H2	0.8600	C10—H10B	0.9700
N3—C1	1.3227 (19)	C11—C12	1.518 (2)
N3—H3A	0.8600	C11—H11A	0.9700
N3—H3B	0.8600	C11—H11B	0.9700
O1—C6	1.3665 (19)	C12—C13	1.513 (3)
O1—C9	1.432 (2)	C12—H12A	0.9700
C2—C3	1.453 (2)	C12—H12B	0.9700
C2—H2A	0.9300	C13—C14	1.517 (2)
C3—C8	1.387 (2)	C13—H13A	0.9700
C3—C4	1.402 (2)	C13—H13B	0.9700
C4—C5	1.377 (2)	C14—C15	1.512 (3)
C4—H4	0.9300	C14—H14A	0.9700
C5—C6	1.390 (2)	C14—H14B	0.9700
C5—H5	0.9300	C15—C16	1.515 (3)
C6—C7	1.389 (2)	C15—H15A	0.9700
C7—C8	1.383 (2)	C15—H15B	0.9700
C7—H7	0.9300	C16—H16A	0.9600
C8—H8	0.9300	C16—H16B	0.9600
C9—C10	1.511 (3)	C16—H16C	0.9600
			
C2—N1—N2	114.83 (13)	C9—C10—H10A	108.7
C1—N2—N1	121.23 (12)	C11—C10—H10B	108.7
C1—N2—H2	119.4	C9—C10—H10B	108.7
N1—N2—H2	119.4	H10A—C10—H10B	107.6
C1—N3—H3A	120.0	C10—C11—C12	113.38 (15)
C1—N3—H3B	120.0	C10—C11—H11A	108.9
H3A—N3—H3B	120.0	C12—C11—H11A	108.9
C6—O1—C9	117.89 (14)	C10—C11—H11B	108.9
N3—C1—N2	117.74 (13)	C12—C11—H11B	108.9
N3—C1—S1	123.14 (12)	H11A—C11—H11B	107.7
N2—C1—S1	119.09 (11)	C13—C12—C11	114.97 (15)
N1—C2—C3	123.26 (14)	C13—C12—H12A	108.5
N1—C2—H2A	118.4	C11—C12—H12A	108.5
C3—C2—H2A	118.4	C13—C12—H12B	108.5
C8—C3—C4	118.26 (14)	C11—C12—H12B	108.5
C8—C3—C2	118.79 (14)	H12A—C12—H12B	107.5
C4—C3—C2	122.94 (15)	C12—C13—C14	113.91 (15)
C5—C4—C3	120.37 (15)	C12—C13—H13A	108.8
C5—C4—H4	119.8	C14—C13—H13A	108.8
C3—C4—H4	119.8	C12—C13—H13B	108.8
C4—C5—C6	120.57 (15)	C14—C13—H13B	108.8
C4—C5—H5	119.7	H13A—C13—H13B	107.7
C6—C5—H5	119.7	C15—C14—C13	114.88 (16)
O1—C6—C7	124.47 (15)	C15—C14—H14A	108.5
O1—C6—C5	115.77 (15)	C13—C14—H14A	108.5
C7—C6—C5	119.76 (15)	C15—C14—H14B	108.5
C8—C7—C6	119.26 (15)	C13—C14—H14B	108.5
C8—C7—H7	120.4	H14A—C14—H14B	107.5
C6—C7—H7	120.4	C14—C15—C16	113.71 (16)
C7—C8—C3	121.78 (15)	C14—C15—H15A	108.8
C7—C8—H8	119.1	C16—C15—H15A	108.8
C3—C8—H8	119.1	C14—C15—H15B	108.8
O1—C9—C10	107.53 (17)	C16—C15—H15B	108.8
O1—C9—H9A	110.2	H15A—C15—H15B	107.7
C10—C9—H9A	110.2	C15—C16—H16A	109.5
O1—C9—H9B	110.2	C15—C16—H16B	109.5
C10—C9—H9B	110.2	H16A—C16—H16B	109.5
H9A—C9—H9B	108.5	C15—C16—H16C	109.5
C11—C10—C9	114.12 (15)	H16A—C16—H16C	109.5
C11—C10—H10A	108.7	H16B—C16—H16C	109.5
			
C2—N1—N2—C1	175.56 (13)	O1—C6—C7—C8	−179.39 (14)
N1—N2—C1—N3	−4.3 (2)	C5—C6—C7—C8	0.7 (2)
N1—N2—C1—S1	174.08 (10)	C6—C7—C8—C3	−0.9 (2)
N2—N1—C2—C3	177.06 (12)	C4—C3—C8—C7	0.2 (2)
N1—C2—C3—C8	−177.92 (14)	C2—C3—C8—C7	179.72 (13)
N1—C2—C3—C4	1.6 (2)	C6—O1—C9—C10	−178.39 (13)
C8—C3—C4—C5	0.7 (2)	O1—C9—C10—C11	−73.0 (2)
C2—C3—C4—C5	−178.83 (13)	C9—C10—C11—C12	−178.21 (16)
C3—C4—C5—C6	−0.8 (2)	C10—C11—C12—C13	176.73 (17)
C9—O1—C6—C7	−6.4 (2)	C11—C12—C13—C14	−179.85 (15)
C9—O1—C6—C5	173.52 (14)	C12—C13—C14—C15	179.08 (16)
C4—C5—C6—O1	−179.76 (13)	C13—C14—C15—C16	−179.20 (17)
C4—C5—C6—C7	0.1 (2)		
